# Unsupervised machine learning method for indirect estimation of reference intervals for chronic kidney disease in the Puerto Rican population

**DOI:** 10.1038/s41598-023-43830-3

**Published:** 2023-10-11

**Authors:** Julian Velev, Jack LeBien, Abiel Roche-Lima

**Affiliations:** 1 Department of Physics, University of Puerto Rico, San Juan, PR 00925-2537 USA; 2 Abartys Health, San Juan, PR 00907-3913 USA; 3 Center for Collaborative Research in Health Disparities - CCHRD, RCMI Program, Medical Science Campus, University of Puerto Rico, San Juan, PR 00936-5067 USA

**Keywords:** Chronic kidney disease, Population screening, Computational models, Machine learning

## Abstract

Reference intervals (RIs) for clinical laboratory values are extremely important for diagnostics and treatment of patients. However, the determination of these ranges is costly and time-consuming. As a result, often different unverified RIs are used in practice for the same analyte and the same range is used for all patients despite evidence that the values are gender, age, and ethnicity dependent. Moreover, the abnormal flags are rudimentary, merely indicating if a value is within the RI. At the same time, clinical lab data generated in the everyday medical practice contains a wealth of information, that given the correct methodology, can help determine the RIs for each specific segment of the population, including populations that suffer from health disparities. In this work, we develop unsupervised machine learning methods, based on Gaussian mixtures, to determine RIs of analytes related to chronic kidney disease, using millions of routine lab results for the Puerto Rican population. We show that the measures are both gender and age dependent and we find evidence for normal age-related organ function deterioration and failure. We also show that the joint distribution of measures improves the diagnostic value of the lab results.

Modern medicine has become heavily reliant on laboratory results for diagnostics. The importance of the interpretation of those results has increased proportionally. Reference intervals (RIs) are a key concept developed to aid the diagnostic interpretation of lab results^1–4^. RIs were introduced to describe the dispersion of analyte values in healthy individuals^3,5^, and it is taken to be the interval of values containing 95% of the individuals^2^. RIs were later adopted by the healthcare community and standardized by the International Federation of Clinical Chemistry (IFCC) and the Clinical Laboratory and Standards Institute (CLSI) who published standards and guidelines of how to establish these intervals^6^.

Traditionally RIs have been obtained by measuring the analyte values in *healthy* populations specifically chosen for that purpose. This *direct* method has severe limitations. It is established that a sample of at least 120 persons is needed for each population and analyte^6^, which makes it prohibitively costly and time-consuming to establish and revise those for most organizations. Additionally, medical experts are needed to separate the healthy from unhealthy individuals. The difficulty of establishing and revising RIs has wide-ranging practical consequences that negatively affect population health. In particular, RIs used in practice are one-size-fits-all, i.e., not gender, age, or ethnicity specific. In the context of ethnicity, the default RIs are primarily based on European/American Caucasians, despite the majority of the global population being from other ethnic groups. At the same time, a number of studies highlight the influence of racial factors on reference ranges^7–12^. For example, substantial discrepancies were identified in nearly all 38 laboratory tests examined across various racial and ethnic groups^13^. Many cardiometabolic measures, such as hemoglobin and total protein, showed significant differences in Hispanic populations compared to others. Another study of 11,254 patients self-identifying on racial or ethnic groups found substantial variations in creatinine levels across the groups^14^.

Alternative *indirect* methods to infer RIs from unlabeled data^15–17^ have emerged due to the availability of large datasets that are produced during the day-to-day medical practice. The use of these data can resolve the issue with the scarcity and the cost associated with the direct method, but at the tradeoff of the lack of diagnostic labels and no control over the sample taking conditions. Thus, to make use of these datasets, robust algorithmic and/or statistical methods are needed to separate healthy from pathological results. Most of the early methods assume that the healthy results are normally distributed, and the pathological results are overlayed on the normal distribution^18,19^. They attempt to infer the normal component of the distribution of the results and obtain the RI from its mean and standard deviation. More recently improved indirect statistical methods have emerged^20–22^, which also treat skewed results distributions making use of logarithmic or Box-Cox transforms^23^. Tools for evaluating the performance of different indirect methods have also been developed^24^.

In this work, we propose a different indirect method for establishing RIs based on unsupervised machine learning (ML). Unsupervised ML methods are designed to work with unlabeled data and learn existing pattens form the data itself. As such, they are superior to parametrized algorithmic and statistical models because they make minimal assumptions about the data and are not constrained to a predefined model. We use a Bayesian Gaussian mixture method to separate the main mode of the distribution from satellite modes caused by age-related chronic conditions.

We apply this novel methodology to determine RIs of metabolic lab results for the Puerto Rican population. Puerto Rico, with a population of approximately 3.2 million people^25^, faces healthcare disparities and challenges, including limited access to services, financial strains, and a high prevalence of cardiometabolic conditions. In this study we focus on a couple of analytes—serum creatinine and blood urea nitrogen (urea, BUN), that are widely used for the diagnosis of chronic kidney disease (CKD)^26,27^. CKD is a condition of progressive loss of kidney function ultimately resulting in the need for renal replacement therapy (dialysis or transplantation)^27^. CKD has come into prominence because the main risk factors for developing it are diabetes, hypertension, and cardiovascular disease^28^, and the prevalence of these conditions has increased dramatically in recent years. According to the Centers for Disease Prevention (CDC) approximately 15% of the US population and 38% of the population older than 65 suffer from medium to advanced CKD resulting in more than 87 billion dollars cost annually in Medicare alone^25^.

## Data

The dataset is supplied by Abartys Health, a health data and analytics company working in the Puerto Rican market. The source of clinical lab results are laboratories in Puerto Rico. The data provided is de-identified by removing all personally identifiable information (PII) such as names, dates of birth, and addresses. Nevertheless, separate individuals can be distinguished by a unique person ID within the dataset, in such a way that the sequence of lab results for the same individual can be followed. Non-PII demographic information, such as age and gender, are available. Geographical location is also available in the form of a postal code. Each lab result is identified by the Logical Observation Identifiers Names and Codes (LOINC)^29^ code of the result and the timestamp of when the sample was taken. The result consists of a value and unit. Abartys Health uses proprietary technology to consolidate results from different labs by mapping the local service IDs of the labs to LOINC codes. It also normalizes and standardizes the values and the units of the readings providing a dataset that is analysis ready.

The clinical measures routinely used in diagnosing CKD are creatinine and urea in blood, as well as albumin and creatinine in urine^27^. The diagnostic value of those measures come from their metabolism. Creatine is a precursor for adenosine triphosphate (ATP), which is the primary energy source of cells. As such it is found in cells with high energy demand and about 95% of it is in the skeletal muscle^30^. Creatine is spontaneously degraded to creatinine in a non-enzymatic reaction. Creatinine then is transported in the blood to the kidneys where it is excreted by glomerular filtration. This increased levels of creatinine in the blood are a sign of impaired kidney function. Based on the level of creatinine in the blood, an estimated glomerular filtration rate (eGFR) is often calculated, which also corrects for factors such as age, sex, and race^31^. Urea is primarily produced during the breakdown of proteins in the liver which produces ammonia as a byproduct. Ammonia is subsequently converted in the liver to the less toxic urea and transported via the bloodstream to the kidneys to be excreted^32^. Thus, the amount of urea in the blood is indicative of the efficiency of the renal clearance.

The data for serum creatinine and urea obtained covers the four-and-a-half-year period between 2019 and mid 2023. The summary of the data is given in Table 1. For both measures, which are part of the routinely performed comprehensive metabolic panel (CMP)^33^, more than 4 million lab results are available of which about 41% are male and 59% female. It is common in real-world data for women to seek medical advice more often than men. The data covers about 1.3 million unique persons which represents about 40% of the Puerto Rican population of roughly 3.2 million^34^. For both analytes the median number of tests per person is 2.0 which is consistent with the frequency of an annual screening test. The average is 3.2 tests per person, which indicates that some persons the sample have a large number of tests.

**Table 1:** Number of results per analyte with demographic information.

Name	LOINC	Results	Male (Unique)	Female (Unique)
Creatinine	2160–0	4,252,381	1,734,379 (554,536)	2,518,002 (761,370)
Urea	3094–0	4,225,173	1,723,413 (554,569)	2,501,760 (761,038)
Creatinine & Urea		4,212,376	1,718,377 (553,511)	2,493,999 (759,720)

We are also interested in the joint distribution of creatinine and urea which is produced by joining the datasets on the unique person ID and date of observation. Since these tests are performed usually as a part of the CMP screening panel^33^, the vast majority of persons have both quantities measured on each lab visit. Thus, the joint data set also has more than 4 million results as shown in Table 1.

## Methods

Most ML applications make use of supervised ML algorithms, where a vector of features ${\varvec{X}}$ is matched to a label or a number ${\varvec{y}}$ for classification or regression methods respectively. The predictive power of these methods is contingent of the availability of a large, labeled training sets. For clinical lab data such a label could be the diagnostic code for the patient as provided by a medical expert. The availability of such labeled data is very limited, and the cost of procuring is very high. At the same time, unlabeled, real-world clinical lab result are continuously produced in large quantities. The challenge of using such data is in the separation of healthy from abnormal results. This is precisely what unsupervised ML methods are intended to do, namely, find patterns in unlabeled data.

In this project we use Gaussian Mixture Model (GMM) and Bayesian Gaussian Mixture Model (BGMM)^35^, to extract the principal Gaussian for the healthy population. GMM models start from K-Means clustering but have the added advantage of being able to apply additional statistical constraints on the data. This gives more flexibility to the shape and size of the clusters they can capture. The GMM assumes that a probability distribution of a multi-dimensional random variable ${\varvec{x}}$ can be approximated by a linear combination of Gaussian distributions

$$p\left({\varvec{x}}\right)=\sum_{k=1}^{N}{a}_{k}{N}_{k}\left({\varvec{x}}|{\mu }_{k},{\Sigma }_{k}\right)$$

where ${N}_{k}$ is the multinormal distribution with mean ${\mu }_{k}$ and covariance matrix ${\Sigma }_{k}$. In GMM the weights ${a}_{k}$ as well as the mean ${\mu }_{k}$ and covariance ${\Sigma }_{k}$ are determined using an Expectation–Maximization (EM) optimization algorithm that maximizes the log-likelihood in the parameter space. The main difference of BGMM is that it uses variational inference (VI). We use the scikit-learn implementation of GMM and BGMM^36–38^.

The primary parameter in these methods is the number of Gaussians within the mixture. In our approach, we employ a two-Gaussian mixture. The first Gaussian serves to capture the central healthy distribution, while the second Gaussian approximates the tails of the distribution, accounting for pathological outcomes. In principle, direct methods have the potential to aid in selecting this parameter by revealing the distinct contributions of various pathological conditions to the overall distribution. The outcome of this estimation process yields the principal Gaussian distribution, characterized by its mean ($\mu$) and covariance ($\Sigma$). This distribution subsequently forms the basis for establishing RIs. The weight assigned to the second Gaussian quantifies the proportion of pathological outcomes within the dataset.

The assessment of model estimation error is achieved through a bootstrapping procedure. This involves resampling the data with replacement to generate random subsets, each comprising a fraction of the original dataset. Subsequently, the RIs are computed within each gender-age segment, and the mean and standard deviation of the RIs are calculated across all subsets. This approach provides a robust measure of model estimation error by accounting for the variability within the dataset.

### Ethical approval

The laboratory test results were retrieved from laboratory information systems serving the clinical laboratories in Puerto Rico. As stipulated by the US Code of Federal Regulation CFR 46.104(d), The analysis of test results does not require patients’ explicit informed consent if the identity of the human subjects cannot readily be ascertained directly or through identifiers linked to the subjects. The use of the datasets has been reviewed and approved by the Institutional Review Board of the office of Human Research Subjects Protection at the University of Puerto Rico—Medical Sciences (reference number 2301072914).

## Results

We proceed to determine the RIs of the CKD-related metabolic measures. In the process, we seek to find support for the following hypotheses: (i) Organ function is gender dependent; (ii) Organ function is age dependent, in particular, organ function deteriorates with age; (iii) Joint diagnostic value of metabolic measures is greater than of them individually.

**Figure 1:**
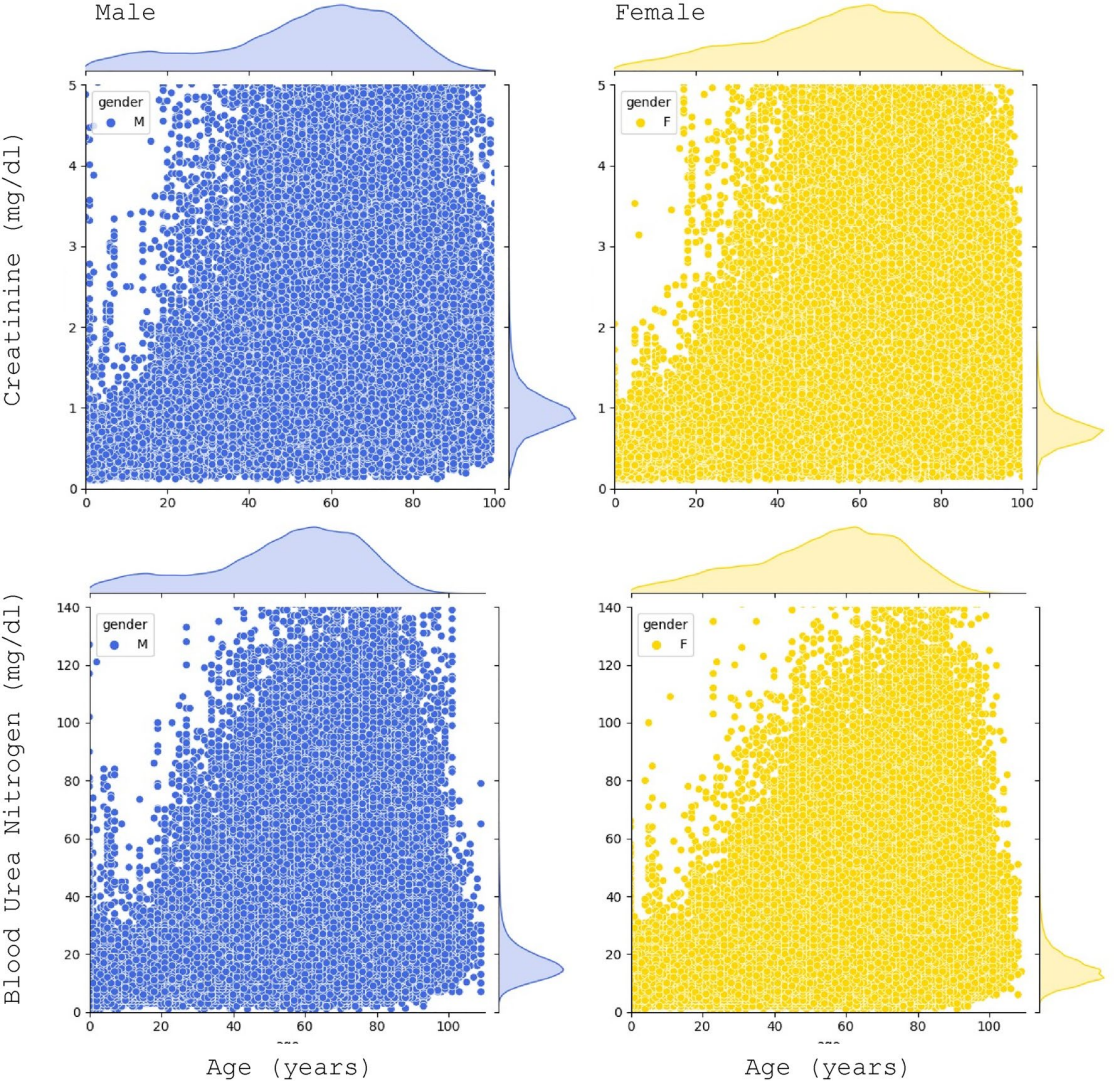
Serum creatinine (top) and blood urea nitrogen (bottom) values vs age for each gender. Population distribution by age is shown in the top margins of the graphs and the distribution of the values in the right margins.

We start by considering the CKD-related metabolic measures individually. The distribution of the result values is show in Fig. 1. In terms of age, the distribution of the population is biased towards older individuals, which is common in real-world data because older individuals are more likely to face health issues and monitor their health. At this stage, the data undergoes an initial outlier removal process, primarily guided by clinical considerations and data inspection. We apply the assumption that readings exceeding 25 mg/dl for creatinine and 150 mg/dl for urea represent potential errors, likely stemming from inaccurately entered values or incorrect units.

From the result distributions in the right margins of Fig. 1, we observe that both the creatinine and urea value distributions for the whole population appear Gaussian-shaped and narrow, although, there are a substantial number of outliers in the data. That means that the healthy individuals’ results are closely clustered together, which is consistent with the idea that normal organ function will produce similar specifications with relatively small variations coming from gender, age, body size, and genetics (ethnicity). To account for these variations, we can apply the methodology for each gender and age segment separately. Unfortunately, information about the body size (height and weight) and ethnicity is not available, which will contribute to some variation within each segment. Additional variation could come from uncontrollable factors, such as the time of the day the sample was taken, time since last meal or exercise, etc. Nevertheless, we expect the healthy results to be clustered together and the pathological results to be in some sense “overlayed” on top of that cluster.

Thus, we start with the data segmentation by gender and age. Since sufficient data is available, the age bins can be a single year. Within the working age range (18–65 years), there are on average over 20,000 results per segment for males and 30,000 for females and no less than 9,500 in each segment in both the creatinine and urea datasets. Additional information into the population distribution and statistics regarding the number of results is given in the supplement.

Then we apply the methodology in each segment separately. The procedure is as follows: (i) segment the data on gender and age; (ii) in each segment, perform a ML Gaussian mixture method to separate the main distribution from the tail; (iii) from the main distribution obtain the low and the high value of the RI; and (iv) interpolate the RI limits to quantify the age and gender dependence and test the hypotheses.

**Figure 2:**
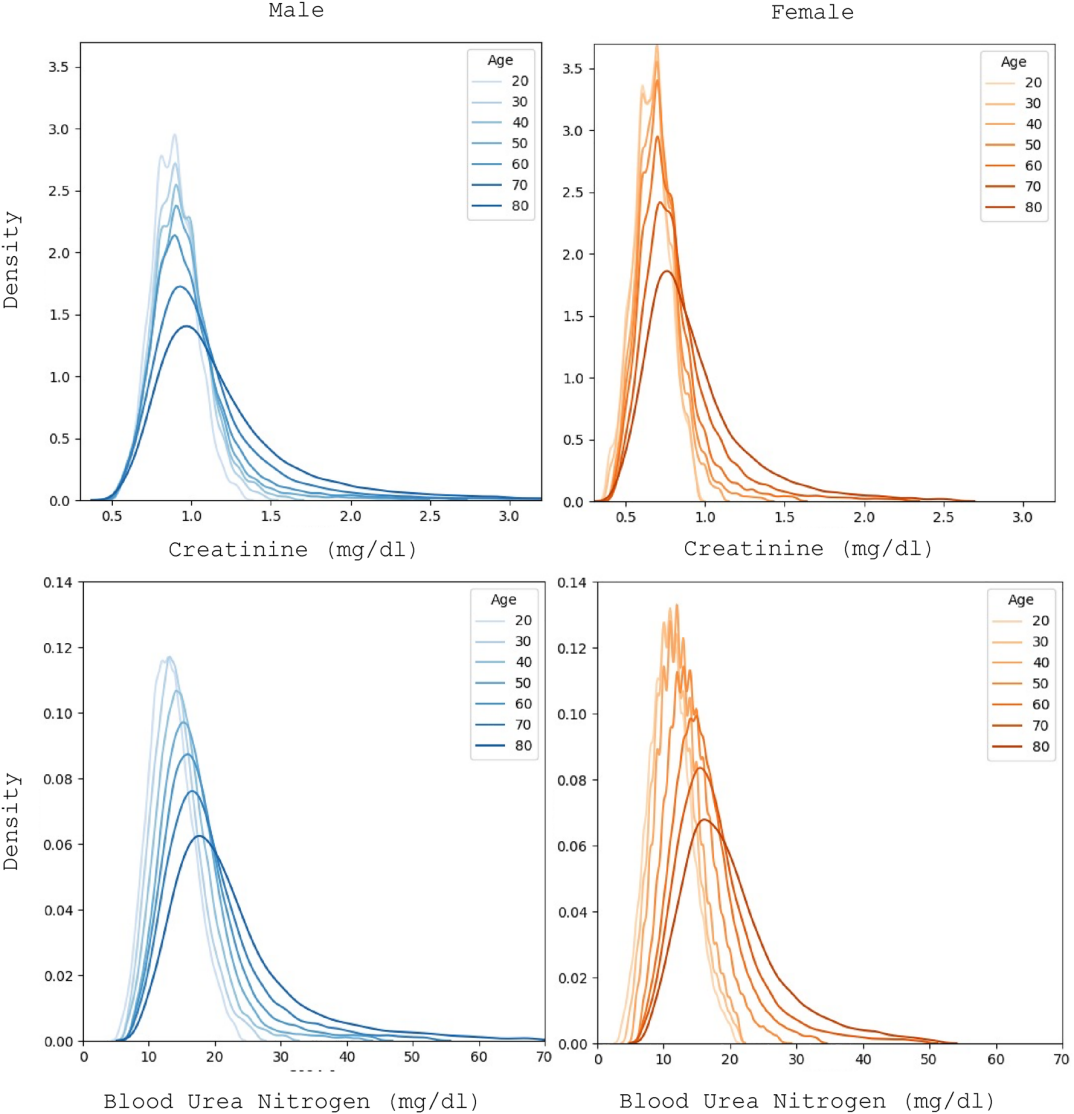
Creatinine (top) and blood urea nitrogen (bottom) distribution of results for different gender and age segments.

A sample of the result distributions in the different segments is shown in Fig. 2 for both creatinine and urea. There are several characteristics of the distributions that are immediately obvious. The low value for all individuals is essentially the same non-zero value, which is consistent with the idea that well-functioning kidneys excrete all the creatinine and urea and the amount of these compounds detectable in the blood is the one being transported at that moment from the muscles (creatinine) or liver (urea) to the kidneys. However, the high values, and thus the mean and width of the distribution, varies dramatically with gender and age.

For creatinine the mean of the distribution for males is higher than for females which could be attributed to the larger body size (and therefore muscle mass)^39^. For urea there is the same trend although less pronounced. The more interesting feature though is the change in the width of the distribution which indicates a clear organ deterioration with age. Young individuals of both genders have very narrow, Gaussian distribution of both creatinine and urea. With age the distribution widens and develops a thick tail towards higher values. Given the evidence that a normal working organ produces a normal distribution, we can assume that the effect of ordinary wear-and-tear simply widens the distribution, and the tail is due to the development of chronic conditions in the aging population.

We can also consider the problem of the shape of the healthy distribution from another perspective. Creatinine is one of the metabolic measures best correlated with body weight. There is evidence that height and weight in the population under normal conditions are normally distributed, however, overweight and obesity result in a thick tail in the weight distribution^40^. Similarly, it has been shown that the distribution of liver enzymes in a lean population is close to Gaussian, whereas a population including the overweight and obese will be markedly skewed^41^.

Thus, the distribution of creatinine and urea values look like a normal distribution with fat tails. We apply the BGMM method in each segment with two components to account for the principal Gaussian and the tail resepectively. Graphs of the decomposition for selected ages are given in the supplement. For creatinine the weight of the outlier component gradually increases from 1% in 20-year-olds to more than 11% in 90-year-olds. While for urea, the weight of the outliers increases from 1 to 16% in the same age interval. The outlier fraction for each population segment is shown in Supp Fig. 1.

**Figure 3:**
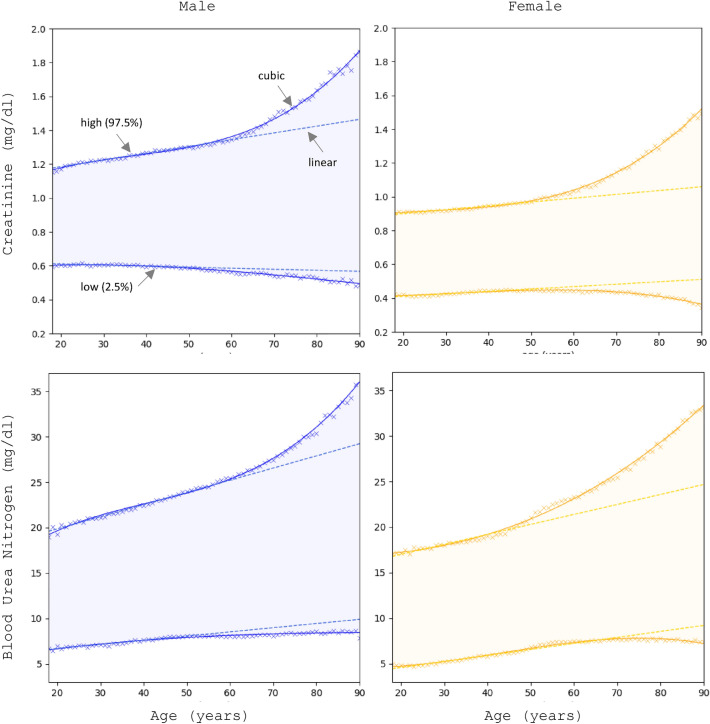
Creatinine (top) and blood urea nitrogen (bottom) reference intervals vs gender and age. Upper and lower limit are calculated as the 2.5 and 97.5 percentile of the distribution of the healthy. Data points for each age are shown with the x symbol. Linear and cubic fits to the data are indicated with solid and dashed lines respectively.

Then from the principal Gaussian we take the 2.5 and 97.5 percentile values as the low and high limits of the RI for that segment. The results for the high and low values for both genders are shown in Fig. 3 as a function of age. As we can see the RI is strongly gender and age dependent. First, we observe that for both measures, the upper limit of the RI for males is higher than for females due to the difference in body size. For creatinine, in the adult population (20–60 years) the male’s RI limits are on average 32% higher than women’s which is consistent with the difference in skeletal muscle mass between the genders^39^. For urea the high limit of the RI is about 16% higher in men than in women.

Second, for both measures there is a very strong age dependence. The RI widens with age, where for adults (20–60) the rate of interval widening is very well approximated by a line. The liner fits are shown in Fig. 3 as dashed lines. The corresponding linear regression coefficients are given in Table 2 for the high RI limit. The liner fit is excellent with R^2^ values above 0.97 and the p-values indicate that the coefficients are statistically significant. We surmise that this regime corresponds to normal wear and tear on the organ. Based on the creatinine graph, the decrease of organ function can be read off the slope of the high limit of the interval and it is on average 0.34%/year. That means that in 10 years adults lose about 3.4% of the kidney function and throughout their adulthood (20–60) about 15% of kidney function. Based on the urea graph, the deterioration of the filtering of urea is even more dramatic, a yearly decrease of 0.67%/year and a staggering 32% over adulthood.

**Table 2:** Linear regression coefficients for the high range limit of creatinine and urea for both genders. Linear fit is performed on the interval of values between ages 20–60 for creatinine and 20–50 for urea.

	Male			Female		
	Slope	Intercept	R^2^	Slope	Intercept	R^2^
Creatinine	0.00387	1.108239	0.99	0.00298	0.83276	0.97
Urea	0.13385	17.18661	0.99	0.10959	14.8156	0.96

Furthermore, the deterioration trend becomes non-linear (accelerating) for elderly persons (> 60) where the graphs are well fitted by a cubic polynomial. The coefficients of the cubic regression are given in the supplement. The transition between linear and accelerated organ function deterioration could be labeled as the onset of organ failure. This is probably due to negative feedback where decreased kidney function damages other organs and, in turn, the increased amount of waste products accelerates the kidney damage.

The low limits remain relatively stable. Since these are waste compounds there is no low limit of those compounds necessary for the organism to function. In the ideal case, the only detectable amounts in the blood would be the quantity just produced but not yet reached the kidneys. For creatinine the minimum quantity seems to be between 0.4 and 0.6 mg/dl, for urea 5.0–9.0 mg/dl. The apparent decrease the low limit of creatinine may be due to the overall decrease of creatinine production as the amount of skeletal muscle decreases in the older population.

**Figure 4:**
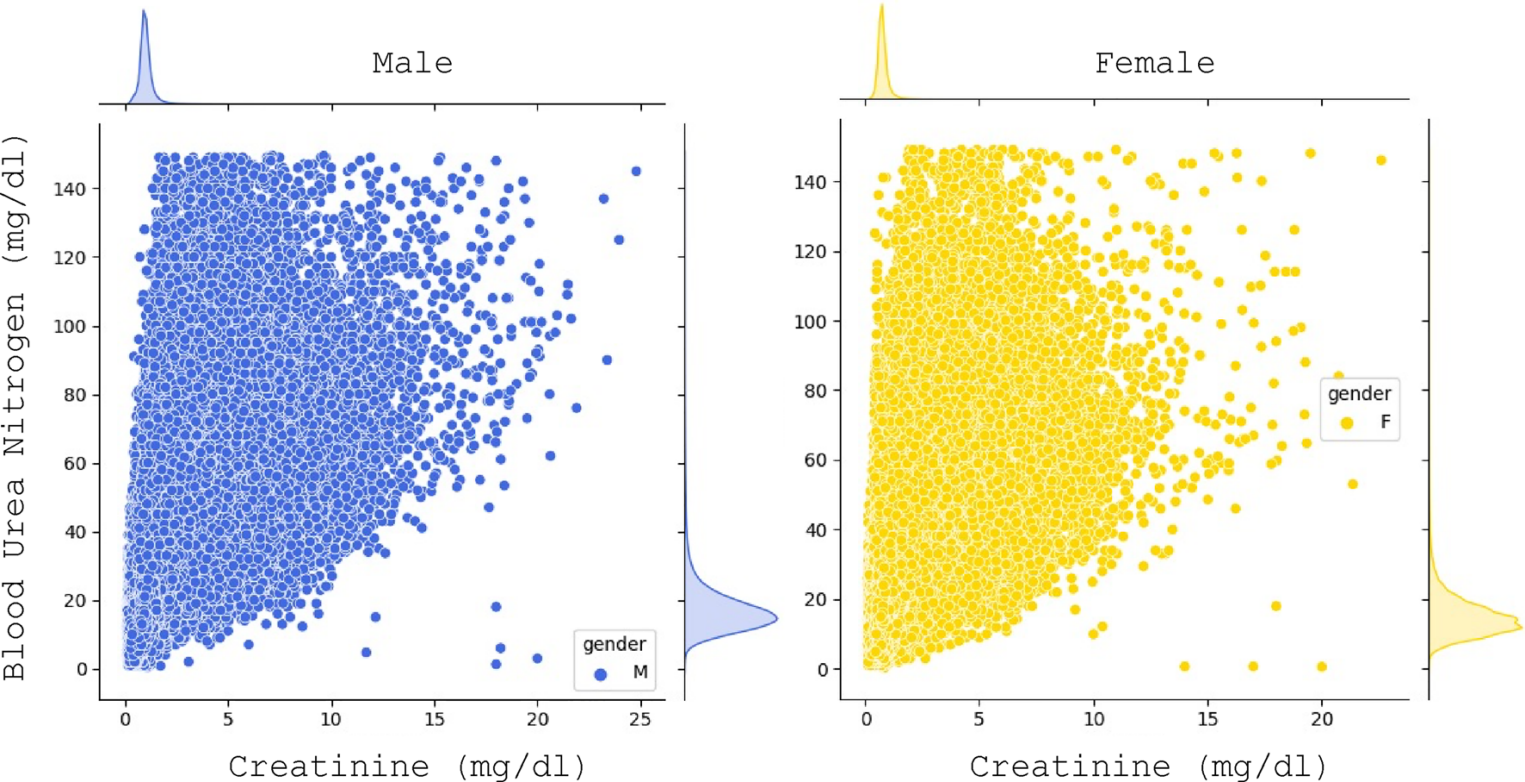
Creatinine vs blood urea nitrogen for male (left) and female (right). The distribution of the individual analytes is shown in the top and right margins.

In clinical practice the values of creatinine and urea are separately labeled as normal or abnormal by the lab and it is left to the physician to make a judgement about kidney function based on these values. However, it could be argued that the joint distribution of the two measures together has greater diagnostic value. Therefore, we consider the joint distribution of creatinine and urea by joining the two datasets and essentially obtain a dataset with all persons who had both quantities measured at the same time. The two-dimensional (2D) distribution of the results is shown in Fig. 4.

We can see from the margin plots that the distribution of the individual analytes is Gaussian-like and narrow, but we have a substantial number of outliers. Fortunately, the ML methodology we have developed is applicable to any number of dimensions. Thus, we follow the same workflow as for the 1D distributions. First, we segment the data into separate groups for each gender and age. Then, in each segment we apply the BGMM method with two components to capture the principal Gaussian and the tail, or rather a “skirt”, of the distribution in 2D. The result of the BGMM method for each gender and age is a multinormal distribution, binormal in this case, with a given mean and covariance matrix.

What is not readily generalizable is the RI itself, which is a strictly 1D concept. To generalize it we can think of the RI as the interval around the mean of the distribution that contains 95% of the results. Thus, in 2D this would be the contour around the mean of the distribution that encircles 95% of the results. We can calculate it by integrating the distribution starting from the mean outwards.

**Figure 5:**
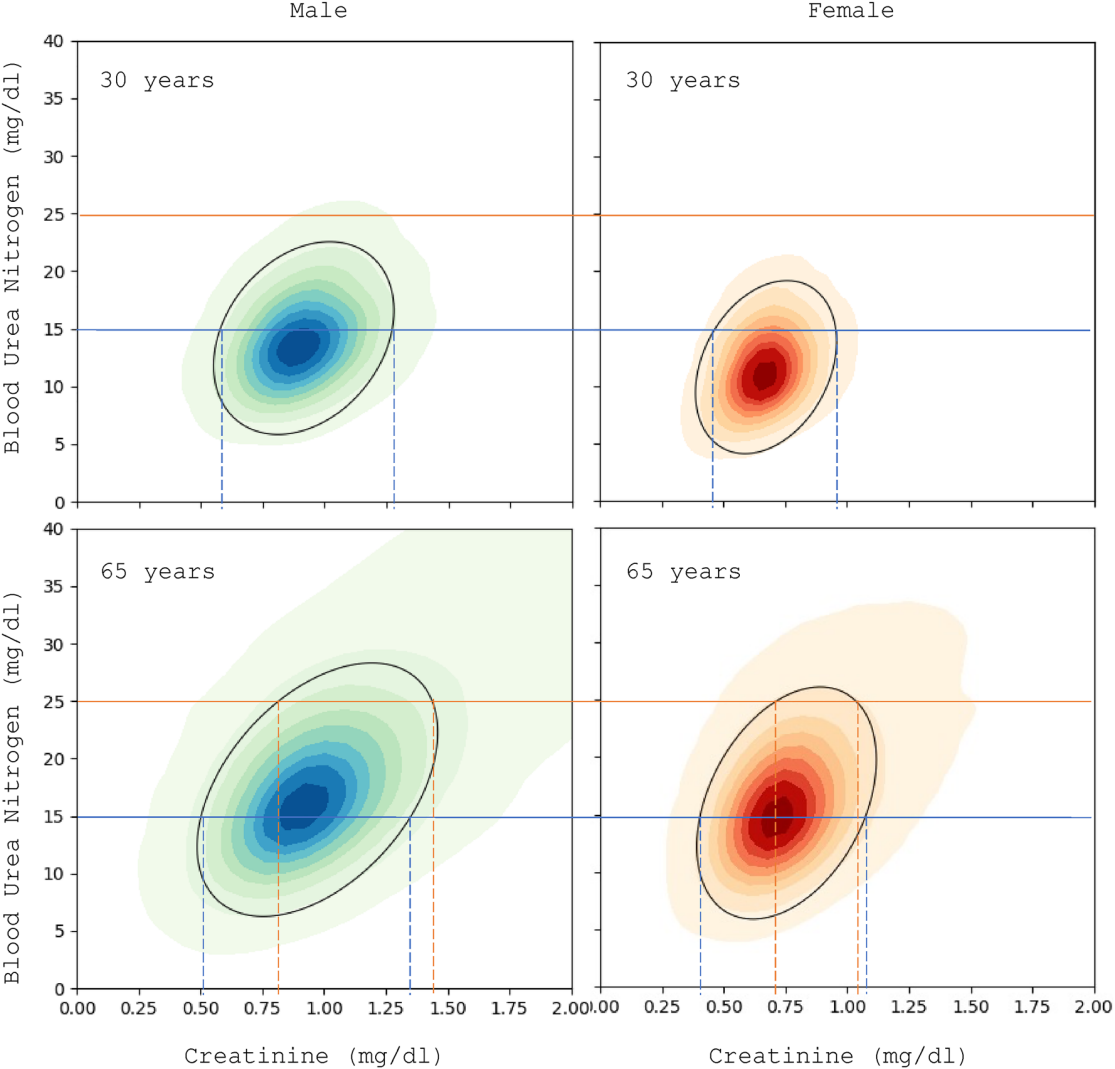
Joint distribution for creatinine and urea for both genders and two age groups, 30- and 65-year-old. The 95% contour is indicated on the graphs as a black ellipse. Hypothetical reference intervals for creatinine are shown for two different levels of urea 15 and 25 mg/dl with blue and red lines.

The joint distributions of creatinine and urea for both genders and two different age groups are shown in Fig. 5. The distributions are strongly gender and age dependent as it could be expected from the results for the 1D distributions. The mean of the distribution for males is higher and to the right than for females due to the differences in body size. With age the distribution becomes wider and less symmetric developing a “skirt” for larger values due to the development of chronic conditions. The BGMM method with two components picks up the principal binormal distribution. By integrating the distribution starting from the mean outward, we obtain the 95 percentile contours which are indicated as ellipses on the figure.

## Discussion

There are several points to be made based on these results. The first observation is intimately related to the definition of the terms “healthy” or “normal” values. What defines *normal* or *healthy* is that where the majority of the population can be found. The subtlety is in defining the population. On the distributions shown in Fig. 2, we could define *perfectly healthy* as being in the 95% of the distribution of *young individuals of the same gender,* after the body has fully developed and without any chronic or congenital illnesses. In this case, the organs function as well as they will ever do. According to this definition, however, most older adults would appear unhealthy. Thus, it is better to define *healthy* as being within the 95% of the mean of the distribution of *individuals of the same gender and age*. This definition takes into account the *normal* organ wear and tear and only flags as abnormal cases where the individual has results anomalous compared to his age group.

In that relation, we propose that there are two reasons for organ deterioration: (i) normal age-related, due to normal wear and tear, and (ii) pathological, due to acute or chronic disease. At least, for creatinine and urea, the evidence in Fig. 2 suggests that for perfectly healthy individuals the distribution of the results is Gaussian where the variation is due to factors not controlled for such as body size, ethnicity, etc. Further on, there is a very good reason to assume that a linear deterioration due to age-related wear and tear would only broaden the distribution. Thus, a Gaussian decomposition of the results distribution provides us with a principal mode which corresponds to our definition of healthy and satellite modes that fit the tails and that correspond to different pathological conditions, in particular chronic conditions that develop with advancing age. Therefore, the RIs thus obtained account for normal organ deterioration with age, but not for chronic conditions, such as obesity or diabetes.

Furthermore, we can consider the evidence for our hypotheses. First, based on the data in Fig. 3, the hypothesis that organ function depends on gender cannot be rejected. We obtained an estimate of the error by bootstrapping, i.e. estimating the RI limits by using samples of the data selected in random. In this case, we created 100 randoms samples of half the dataset size. RIs are calculated as the mean over the samples, and the error as the standard deviation. We obtained the average estimation error to be 0.02% which makes it too small for the error bars to be visible in Fig. 3. Thus, the male and female RIs are well-separated, beyond any statistical uncertainty.

Second, the hypothesis that organ function deteriorates with age cannot be rejected either. The slope of the RI upper limit estimated from the linear regression of the linear part of the graph for adults (20–60) is statistically different from zero as evident from the p-value of the fit. The coefficients of the fit for the non-linear part of the graph are also statistically significant as shown in the supplement.

Third, based on the data plotted in Fig. 5 for the joint distribution of the analytes, it is immediately clear that the joint distribution has higher diagnostic value than the individual distributions. If we focus on the lines drawn in the graph, we can see that the RI of creatinine is strongly dependent on the value of urea. For example, for a 65-year-old female, who has a value of urea of 15 mg/dl, the RI for creatinine would be approximately (0.4, 1.1) mg/dl. But for the same female with a reading for urea of 25 mg/dl, the acceptable values for creatinine would be in a much narrower range around (0.7, 1.0) mg/dl. If we compare with a 30-year-old female with a urea reading of 15 mg/dl, the RI for creatinine is now narrower (0.4, 0.9) mg/dl. And for a urea reading of 25 mg/dl the interval does not exist, i.e. the urea reading alone implies that the result is outside of the 95 percentile range. Quantitatively speaking, in the single-analyte case the RI for creatinine is obtained as being in the 95% of the distribution ${P}_{g,a}\left(c\right)$ which is the probability for a creatinine reading of $c$ parametrized by the gender $g$ and age $a$. In contrast the, in the two-analyte case the RI is obtained from the conditional distribution ${P}_{g,a}\left(c|u\right)$ which is the probability for a creatinine reading of $c$ parametrized by the gender $g$ and age $a$ given a urea reading of $u$. This creatinine RI will be different for each urea reading.

This approach is not limited to the bi-analyte case. A standardized renal test panel^42^, essentially a subset of the comprehensive metabolic panel (CMP)^43^, is routinely used by physicians for general health screening and diagnostics. In addition to creatinine and urea, the panel includes metabolic measures indicative of kidney function such as serum glucose, albumin, and electrolytes. Given that these tests are routinely conducted as a panel, a substantial volume of data is available for constructing the joint distribution of these measures. Consequently, this approach allows us to derive more specific RIs for each measure and to enable the development of a multi-analyte diagnostic model.

In this relation, we can make the point that these results go well beyond the rudimentary idea of RIs. The RI is a strictly 1D concept that implies that the different analyte readings are independent, which is clearly not the case from a physiological perspective. Also, the RI only gives the upper and lower limit, and therefore only a binary value of the abnormal flag (AF)—normal (inside the interval) or abnormal (outside). On the contrary, our analysis results in a distribution. Thus, the AF can be made continuous by giving the percentile in the distribution that a reading is in. Thus, readings that are closer to the center of the distribution can be considered more healthy or less risky, which makes the AF a measure of risk, not merely a flag. In fact, the method to evaluate AF with best diagnostic value should be to pass the reading to a model which would return the percentile of the result accounting for the patient’s gender and age. And there could be also diagnostic panels, based on multiple analytes, which would assign a percentile on the group of readings together.

As an example of how this works, we have created an augmented model for the CKD based on the RIs calculated before. The model parameters are the means and covariances of the RI distributions for creatinine and urea separately and together parametrized by gender and age. The predict function of the model takes the analyte(s) readings and calculates the percentile of the reading in the distribution, by calculating the probability of the reading and the cumulative probability above this probability level. For example, for a 30-year-old male with reading of creatinine of 1.3 mg/dl and urea of 10.0 mg/dl, the model gives that the creatinine result is in the 98% range (abnormal) and the urea result is in the 75% range (normal). The percentile of measuring both of them together is in the 79% range. In other words, although the creatinine result is abnormal both results taken together are within the normal range for this gender/age segment of the Puerto Rican population. More examples are provided in the supplement.

## Conclusion

Overall, we used a big-data, unsupervised ML approach to determine the RIs for the CKD-related analytes serum creatinine and blood urea nitrogen for the Puerto Rican population. Such a large-scale study is unthinkable in the context of the direct methods to determine the RIs. We used unsupervised ML methods to separate the healthy from pathological results. From the data distribution, we determined the upper and lower limit of the RIs as a function of gender and age. We found that the RIs are strongly gender and age dependent, and therefore the use of a single interval in real-world lab reports is misleading.

Even more interestingly, we observed that there is age-related deterioration of organ function due to normal wear and tear which is approximately linear for adults (20–60), after which commences a period of accelerated organ failure likely due to a negative feedback loop between the kidneys and other systems in the body.

We also propose a generalization of the RI and AF concepts to the percentile value of the reading in the distribution of the analyte for the population of the same age and gender. Furthermore, we show that we can construct panels of analytes and assign a percentile value of a set of readings together based on the joint distribution of the analytes. This functionality essentially imitates and quantifies the integration of lab result readings that a medical expert performs in his head based on his knowledge and experience. And as such, it is a step further towards an automated medical diagnostic expert system.

### Supplementary Information

Supplementary Information.

## Data Availability

The data that support the findings of this study were licensed from Abartys Health for the purposes of this study alone and are not publicly available. Data access requests can be addressed to the corresponding author who would relay them to Abartys Health. Reasonable requests for access to the original code used to analyze the data can be directed to the corresponding author.
