# Supplementary material for: Unsupervised machine learning method for indirect estimation of reference intervals for chronic kidney disease in the Puerto Rican population
Source: Sci Rep. 2023 Oct 11;13:17198. doi: 10.1038/s41598-023-43830-3 (PMC10567761; doi:10.1038/s41598-023-43830-3)
Supplement: Supplementary file 1 — Supplementary Information. [file 41598_2023_43830_MOESM1_ESM.docx]

**Supplementary Information for Unsupervised Machine Learning Method for Indirect Estimation of Reference Intervals for Chronic Kidney Disease in the Puerto Rican Population**

Julian Velev,^1,2^ Jack LeBien,^2^ and Abiel Roche-Lima^3^

^1^ Department of Physics, University of Puerto Rico, PR 00925-2537

^2^ Abartys Health, San Juan, PR 00907-3913

^3^ Research Capacity Core, University of Puerto Rico – Medical Sciences, San Juan, PR 00936-5067

**Methods**

We use the scikit-learn implementation of Bayesian Gaussian mixture model (BGMM).^1^ The class sklearn.mixture.BayesianGaussianMixture is initialized with two components and fill covariance as shown in Supp Table 1.^2^ The prior mean is set to be the mean of the original distribution and we use high prior weight concentration. The rationale is that one of the Gaussians captures the healthy distribution which is close to the center of the distribution and the other captures the pathological cases which contribute to the tail.

Supp Table 1: Parameters of the Bayesian Gaussian mixture model.

| **Parameter** | **Value** |
| --- | --- |
| n_components | 2 |
| covariance_type | full |
| mean_prior | sample mean |
| weight_concentration_prior | 1000 |

**Data**

The number of results in each gender and age segment is shown in Supp Figure 1. First, the number of results for creatinine and urea are comparable because these tests are performed as a part of the same panel of tests, namely the renal panel or the comprehensive metabolic panel.^3,4^

Second, the number of results is skewed towards older individuals due to the appearance of health problems with age and the increase of routine checkups performed by the physicians. Around 40 years is we observe an inflection point where the rate of testing significantly increases, until it reaches a maximum around 65 years of age, after which the mortality reduces the number of individuals and hence test results. Females go 47% more often to the labs than males.

Supp Figure 1: Number of lab results in each gender-age segment for serum creatinine (top) and urea (bottom). The fraction of outliers in the sample identified by the Gaussian mixture model is indicated with the red band.

Some basic statistics for the number of results and outliers is shown in Supp Table 2. In the working age range (18-65 years) the average number of results in each segment is more than 20,000 for males and 30,000 for females for both creatinine and urea.

Supp Table 2: Number of test results statistics by gender for the working age population (18-65 years).

|  | **Creatinine** | | **Urea** | |
| --- | --- | --- | --- | --- |
| **Results (#)** | **male** | **female** | **male** | **female** |
| mean | 20,628 | 32,121 | 20,492 | 31,890 |
| min | 9,405 | 14,286 | 9,357 | 14,208 |
| max | 36,830 | 51,620 | 36,462 | 51,239 |

**Results**

We apply the Bayesian Gaussian Mixture model (BGMM) on the analytes’ distributions in each gender and age segment. We use two components – one to capture the main mode of healthy results and one to account for the tail of the distribution that develops due to the development of chronic conditions with age.

Supp Figure 2: Gaussian mixture components superimposed on the creatinine distribution for both genders and several age groups. The fat tail of the distributions is indicated by the red arrow.

The component Gaussians superimposed on the creatinine distribution are shown in Supp Figure 2. It could be seen that in young individuals the distribution of the results is very nearly Gaussian and narrow. With age the principal mode widens due to normal, age-related wear and tear, but also the distribution develops a tail due to the admixture of pathological, very likely chronic conditions. The weight of the tail is obtained from the weight of the second Gaussian. It generally increases with age from less than 1% to more than 10% for creatinine and more than 15% for urea over the whole age range.

Supp Table 3: Outlier statistics by gender for the working age population (18-65 years).

|  | **Creatinine** | | **Urea** | |
| --- | --- | --- | --- | --- |
| **Outliers (%)** | **male** | **female** | **male** | **female** |
| mean | 3.4 | 2.3 | 4.7 | 3.1 |
| min | 0.6 | 0.4 | 1.1 | 0.5 |
| max | 8.5 | 5.9 | 10.6 | 7.9 |

**Reference interval interpolation**

We are interested in the functional behavior of the RI limits. For both analytes there seems to be a linear part, approximately between 20-60 years and a non-linear portion beyond 60 years of age. We perform a least-squares linear regression on the linear part of the graph for both analytes. The functional dependence is

$$y=ax+b$$

where $y$ is the analyte value and $x$ the age. The estimated coefficients of the least squares regression are as follows:

| **Male** | $a$ | $b$ | $R^{2}$ |
| --- | --- | --- | --- |
| Creatinine | 0.00387 | 1.108239 | 0.99 |
| Urea | 0.13385 | 17.18661 | 0.99 |
| **Female** |  |  |  |
| Creatinine | 0.00298 | 0.83276 | 0.97 |
| Urea | 0.10959 | 14.8156 | 0.96 |

We find that the accelerated organ deterioration is very well fit by a cubic polynomial

$$y=ax^{3}+bx^{2}+cx+d$$

where the coefficients for the upper limit of the RI are as follows:

| **Male** | $a$ | $b$ | $c$ | $d$ | $R^{2}$ |
| --- | --- | --- | --- | --- | --- |
| Creatinine | 2.08e-7 | -5.71e-5 | 0.00258 | 0.57341 | 0.99 |
| Urea | 2.32e-6 | -0.00077 | 0.08617 | 5.23624 | 0.99 |
| **Female** |  |  |  |  |  |
| Creatinine | 1.95e-6 | -0.00015 | 0.00516 | 0.84905 | 0.93 |
| Urea | -2.45e-5 | 0.00317 | -0.06150 | 0.06598 | 0.99 |

**Augmented reference intervals and abnormal flags**

Using the calculated individual and joint distributions of the RIs we can create a model to augment the standard RIs and AFs with gender and age specific RIs for the particular population and a continuous AF which indicated the percentile of the result in the distribution. The model parameters are the means and covariances of the RI distributions for creatinine and urea separately and together parametrized by gender and age. The model parameters are stored in files in JSON format to use for augmented RI calculation. Snippets of the model parameter files are show in Supp Figure 3.

Supp Figure 3: Snippets of augmented RI parameter files in JSON format for both the 1D and 2D models.

Examples of the augmented model are given in Supp Table 4 and Supp Table 5 for creatinine and urea respectively.

### Creatinine:

According to CDC, the creatinine the RI is (0.67, 1.17) mg/dl for male and (0.51, 0.95) mg/dl for female.^5^ The abnormal flag is only indicative of whether the value is inside or outside of the range. While the CDC RIs are gender specific, they are not age specific. In our case, the RIs are both gender and age specific. The abnormal flag is the percentile of the result within the population. As shown in Supp Table 4, due to the age dependence, borderline values of creatinine of 1.3 mg/dl for male and 1.0 mg/dl for females are abnormal according to the CDC ranges, but normal for older individuals according to the augmented RI model.

Supp Table 4: Examples of augmented reference intervals for serum creatinine.

| **Gender** | **Age** | **Value** | **Range (CDC)** [mg/dl] | **Abnormal** | **Range (New)**  [mg/dl] | **Abnormal (New)** (< 95%) |
| --- | --- | --- | --- | --- | --- | --- |
| M | 30 | 1.3 | 0.67-1.17 | A | 0.60-1.23 | 98% (A) |
| M | 65 | 1.3 | 0.67-1.17 | A | 0.56-1.39 | 87% (N) |
| F | 30 | 1.0 | 0.51-0.95 | A | 0.43-0.92 | 99% (A) |
| F | 65 | 1.0 | 0.51-0.95 | A | 0.44-1.07 | 88% (N) |

### Urea:

Similarly, the CDC used RI for urea is (6.0, 23.0) mg/dl for both genders. The augmented RIs are both gender and age specific. As shown in Supp Table 5, borderline values of 22 mg/dl for males and 20 mg/dl for females are normal according to the CDC RIs, but abnormal for younger individuals according to the augmented RI model.

Supp Table 5: Examples of augmented reference intervals for serum urea.

| **Gender** | **Age** | **Value** | **Range (CDC)** [mg/dl] | **Abnormal** | **Range (New)**  [mg/dl] | **Abnormal (New)** (< 95%) |
| --- | --- | --- | --- | --- | --- | --- |
| M | 30 | 22 | 6.0-23.0 | N | 7.1-21.1 | 97% (A) |
| M | 65 | 22 | 6.0-23.0 | N | 8.1-26.9 | 70% (N) |
| F | 30 | 20 | 6.0-23.0 | N | 5.2-18.0 | 99% (A) |
| F | 65 | 20 | 6.0-23.0 | N | 7.6-24.2 | 66% (N) |

### Creatinine & urea:

Finally, the diagnostic value of creatinine and urea together is shown in Supp Table 6. The individual measures misdiagnose the 30-year-old male as having CKD (based on creatinine), while the joint measures indicate that the result is normal. Similarly, based on urea alone, the 30-year-old female can be misdiagnosed as having CKD, while the joint measures indicate that the result is not a risk for CKD, but it could be for another condition affecting the liver.

Supp Table 6: Examples of augmented reference intervals jointly for creatinine and urea.

| **Gender** | **Age** | **Creatinine** [mg/dl] | **Urea**  [mg/dl] | **Creatinine** (< 95%) | **Urea** (< 95%) | **Creatinine & Urea** (< 95%) |
| --- | --- | --- | --- | --- | --- | --- |
| M | 30 | 1.3 | 20.0 | 98 (A) | 89 (A) | 90 (N) |
| F | 30 | 0.8 | 20.0 | 69 (N) | 99 (A) | 73 (N) |

1. Pedregosa, F. *et al.* Scikit-learn: Machine Learning in Python. *Journal of Machine Learning Research* **12**, 2825–2830 (2011).

2. scikit-learn 1.3.0. Bayesian Gaussian Mixture Model. https://scikit-learn.org/stable/modules/generated/sklearn.mixture.BayesianGaussianMixture.html.

3. loinc.org. Renal function 2000 panel - Serum or Plasma. *https://loinc.org/24362-6/* (2023).

4. loinc.org. Comprehensive metabolic 2000 panel - Serum or Plasma. *https://loinc.org/24323-8/* (2023).
